# Craniofacial therapy: advanced local therapies from nano-engineered titanium implants to treat craniofacial conditions

**DOI:** 10.1038/s41368-023-00220-9

**Published:** 2023-03-29

**Authors:** Karan Gulati, Chengye Ding, Tianqi Guo, Houzuo Guo, Huajie Yu, Yan Liu

**Affiliations:** 1 The University of Queensland, School of Dentistry, Herston, QLD Australia; 2 Laboratory of Biomimetic Nanomaterials, Department of Orthodontics, Peking University School and Hospital of Stomatology, Beijing, China; 3 National Center for Stomatology & National Clinical Research Center for Oral Diseases & National Engineering Laboratory for Digital and Material Technology of Stomatology & Beijing Key Laboratory of Digital Stomatology & Research Center of Engineering and Technology for Computerized Dentistry Ministry of Health & NMPA Key Laboratory for Dental Materials, Beijing, China; 4 Department of Oral Implantology, Peking University School and Hospital of Stomatology, Beijing, China; 5 Fourth Clinical Division, Peking University School and Hospital of Stomatology, Beijing, China

**Keywords:** Bioinspired materials, Biomimetic synthesis

## Abstract

Nano-engineering-based tissue regeneration and local therapeutic delivery strategies show significant potential to reduce the health and economic burden associated with craniofacial defects, including traumas and tumours. Critical to the success of such nano-engineered non-resorbable craniofacial implants include load-bearing functioning and survival in complex local trauma conditions. Further, race to invade between multiple cells and pathogens is an important criterion that dictates the fate of the implant. In this pioneering review, we compare the therapeutic efficacy of nano-engineered titanium-based craniofacial implants towards maximised local therapy addressing bone formation/resorption, soft-tissue integration, bacterial infection and cancers/tumours. We present the various strategies to engineer titanium-based craniofacial implants in the macro-, micro- and nano-scales, using topographical, chemical, electrochemical, biological and therapeutic modifications. A particular focus is electrochemically anodised titanium implants with controlled nanotopographies that enable tailored and enhanced bioactivity and local therapeutic release. Next, we review the clinical translation challenges associated with such implants. This review will inform the readers of the latest developments and challenges related to therapeutic nano-engineered craniofacial implants.

## Introduction

The craniofacial tissue, consisting of bone, cartilage, muscle, salivary glands, nerve tissue, teeth and the surrounding periodontium, and skin/mucosa,^1^ is prone to numerous diseases, disorders and injuries, including trauma and tumours. To date, the prevalence of craniofacial deformity and the economic and social burden associated with it represents a global treatment challenge. As is shown in The Global Burden of Diseases, Injuries, and Risk Factors Study 2017 (GBD 2017), oral disorders ranked the highest in age-standardised prevalence and incidence globally.^2^ For instance, periodontitis, a chronic inflammatory disease associated with dysbiosis in host-community interaction,^3,4^ contributes to the increasing burden of oral diseases.^2^ From 1990 to 2019, the age-standardised prevalence rate of severe periodontitis increased by 8.44% (6.62%–10.59%) worldwide. In 2019, there were 1.1 billion (95% uncertainty interval: 0.8–1.4 billion) prevalent cases of severe periodontitis globally.^5^ Further, untreated periodontal diseases can progressively deteriorate periodontal attachments and alveolar bone, ultimately leading to tooth loss and oral dysfunction.^6^

Osseointegrated dental implants are now widely applied in the rehabilitation of dentition defects. Nevertheless, the associated complications, such as peri-implant mucositis and peri-implantitis, are considered significant and expanding problems due to the worldwide prevalence of dental implants. Peri-implantitis is now defined as a chronic inflammatory condition induced by a bacterial biofilm in susceptible hosts.^7^ Typical clinical manifestations of peri-implantitis include soft-tissue inflammation and progressive loss of peri-implant bone.^8,9^ The severity of peri-implantitis lesions correlates with the level of submucosal microbial dysbiosis, and when there is increasing bone loss and peri-implant disease, suppuration may follow.^10^ In a study where 458 dental implants from 89 patients were examined, the prevalence of peri-implantitis was 56.6% at the patient level while 27.9% at the implant level.^11^

Another common cause of craniofacial defects is trauma. Seemingly, 69 million individuals suffer from traumatic brain injury (TBI) each year.^12^ Likewise, evidence from GBD 2019 demonstrates that road injury ranked 1st in the top ten causes of global disability-adjusted life-years in the 10–24 and 25–49 age groups.^13^ In China, population-based mortality of TBI is estimated to be ~13 cases per 100,000 people. At the same time, the absolute number of patients with TBI exceeds that of most other countries, posing a considerable burden on society and families.^14^ Specifically, TBI costs the global economy approximately $US400 billion annually, and the burden of TBI from road traffic incidents is increasing.^15^ In addition, traumatic injuries, such as falling injuries in childhood, are the leading cause of unilateral (or bilateral) temporomandibular joint injury, micrognathia and facial asymmetry.^16–18^

Additionally, tumours in the craniomaxillofacial region, whether benign or malignant tumours may cause localised tissue abnormalities. Ameloblastoma is a common benign disease that originates from odontogenic epithelial cells and results in the formation of a severe intraosseous mass that exhibits aggressive biological behaviour with a risk of recurrence.^19^ Resection surgery of the tumour is often performed in patients with ameloblastoma, yet it inevitably leads to a massive tissue defect. Among the malignant tumours, head and neck squamous cell carcinoma (HNSCC) is the sixth most common cancer worldwide.^20^ It has become a significant health challenge attributed to high incidence and mortality, with 890 000 new cases and 450 000 deaths in 2018.^21,22^ It is estimated that the incidence of HNSCC will continue to increase by 30% (~1.08 million new cases annually).^20^ The recent surge in the prevalence of HNSCC is increasingly attributed to the infection of oncogenic strains of human papillomavirus (HPV), primarily HPV-16.^23,24^ Craniofacial malignant tumours are generally treated with surgical resection and supplemented with other treatment methods, such as radiation, chemotherapy, or immunotherapy.^25^ The side effects of conventional tumour therapy include substantial tissue loss and residual bone defect in the craniofacial region,^26^ which usually bring about profound disabilities and decrease patients’ quality of life. Around 50% of HNSCC survivors experience swallowing and speech impairments after radiation therapy.^27^ These sequelae may affect patients in the long term. Surveys have revealed that 68% of HNSCC survivors reported voice problems even 10 years after radiotherapy.^28^

Congenital malformations are another critical cause of the craniofacial region’s soft- and hard-tissue defects. For example, cleft lip and palate, arising in ~1.7 per 1000 newborns and affecting speech, hearing, appearance and psychology, can negatively impact their health and social integration.^29^ Microtia, on the other hand, is believed to represent the mildest form of craniofacial microsomia.^30^ And it usually affects the intrauterine development of the auricle, with a prevalence of 2.06 per 10,000 births.^31^ According to the pattern of malformations, other craniofacial disorders include holoprosencephaly, skull vault malformations (such as craniosynostosis) and malformations of the first and second branchial arches.^32^

**Fig. 1:**
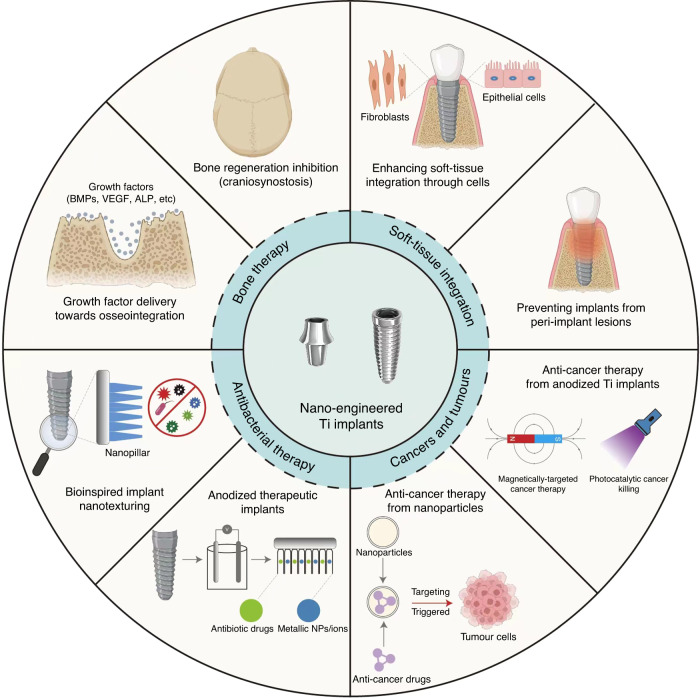
Craniofacial therapy. Craniofacial defects and ailments require extensive local therapeutic administration

Above all, with a wide range of etiology (periodontal diseases, trauma, tumour, infection and congenital malformation), craniofacial defects fall into several categories, including bone, cartilage, soft tissue and tooth defects; and often involve complex tissue defects in clinical practice. These impairments negatively influence the patients’ quality of life by impacting vital oral functions, including chewing, speaking, nutrition and facial aesthetics. Therefore, designing an effective therapeutic strategy for craniofacial defects is critical to reduce the associated health and economic burden. Figure 1 summarizes the various craniofacial conditions that require effective local therapeutic administration.

This review discusses and details the critical advances in craniofacial therapy to alleviate challenges associated with conventional drug administration. Head and neck tumours, craniosynostosis, dental implant failure and poorly osseointegrated implants represent craniofacial defects and conditions that can be treated via local drug-eluting implants. Specifically, electrochemically anodised Ti implants with TiO_2_ nanotubes represent a favourable implant surface modification strategy that can enable customisable local drug delivery treating the abovementioned conditions while providing necessary mechanical support and bioactivity. We look closely at the therapeutic efficacy of such drug-eluting implants to achieve maximum therapeutic action directly inside the craniofacial microenvironment.

## Craniofacial implants and therapeutic challenges

### Craniofacial grafts vs implants

Functional, morphological and aesthetic recovery are the main aims of craniofacial restoration.^33^ Craniofacial bone defects might range in complexity from simple segmental defects to minor periodontal problems. Further, with sufficient underlying bone integrity, soft tissue restoration is straightforward in cases of significant craniofacial abnormalities.^34,35^ There are natural and synthetic options for the biomaterials utilised as craniofacial grafts.^35^ Autologous bone grafting is the gold standard for orthopaedic and cranio-maxillofacial bone reconstruction.^36^ However, the process of autografts will inevitably damage the donor site, increasing the risk of postoperative complications.^37^ Synthetic biomaterials can be resorbable or non-resorbable and vary from biometals to biocomposite materials. Further, synthetic grafts have the benefits of being sterile, consistent in quality, widely available, and low in morbidity.^35^

In conditions where autologous reconstruction is not acceptable, implants, especially non-resorbable implants, have shown great promise in supporting craniofacial repair, including oral, orbital, auricular and midface prothesis.^38^ Implantable medical devices are widely applied in orthopaedics and dentistry to correct or replace tissues. There are around 10 000 000 dental implants inserted worldwide annually.^39^ In the USA, the prevalence of dental implants is expected to climb from 5.7% under the most conservative scenario to 23% under the least optimistic one in 2026, attributed to an aging population.^40^

### Craniofacial implants: integration and infection

Biomaterials like metal-based implants are necessary for improving damaged tissues’ structural and functional integrity in biomedical applications. For craniofacial correction, ideally, the implants must meet the following requirements^41^:

- Biosafety and biocompatibility.
- Osteoinductive or osteoconductive nature.
- Mechanical stability.
- Ease of fabrication and sterilisation.
- Orchestrate both hard- and soft-tissue integration.

Following implant placement, there are two main postoperative complications: infection and inadequate integration. As a result, implant loosening (micro-motion), fracture malunion or nonunion, and implant failure may occur.^42,43^ Further, the growth of intervening soft tissue rather than bone tissue and stress shielding^44^ also dictate the extent of implant integration.

Due to the rising use of biomaterial implants and devices, a significant consequence known as biomaterial-associated infections (BAIs) poses a threat. The prevalence of BAI is typically between 0.5 and 6%.^45^ Bacterial adherence and biofilm formation on the implant surface are the most common causes of implant-associated infection.^43^ *S. aureus* and *P. gingivalis*, the major pathogenic bacteria for orthopaedic osteomyelitis and dental peri-implantitis, respectively, are responsible for biofilm formation on implants.^46,47^ Once the biofilm has been established, the bacteria inside it may be able to fend off the host’s immunological response. Further, antibiotic-resistant bacteria found in biofilms generally have minimum inhibitory concentrations 10–1 000 times higher than those found in planktonic bacteria.^48^ Additionally, microbial infections resulting in biofilm development can result in inflammation at the implant site and impairs osseointegration.^49^

Conventional treatment for bone infection requires the surgical removal of any necrotic bone or prosthetic material and the debridement of inflammatory granulation. Antibiotics administered systemically at a high enough dose to reach the necrotic area and eradicate the infection usually cause systemic toxicity.^50^ Thus, it is imperative to develop efficient local therapeutic delivery strategies.

Besides, a fundamental factor determining the implant’s fate is the race for the surface between different cells and pathogens. The term ‘race for the surface’ was first used by orthopaedic surgeon Anthony G. Gristina in 1987 to describe what would happen to biomaterial implants as BAI developed.^51^ If bacteria win the race, the implant surface will swiftly be covered with a biofilm, and bacterial virulence factors and toxins will interfere with the function of tissue cells. To achieve successful implant osseointegration, it is necessary to balance the competition between bacteria and osteoblasts, i.e., sufficient osteoblast growth while inhibiting bacteria.^43^ Further, timely formation and long-term maintenance of soft-tissue integration at the transmucosal region of dental implants is crucial as it forms a barrier to prevent the ingress of pathogenic bacteria.^52^

### Craniofacial implants: local trauma and inflammation

Another crucial criterion to ensure early stability and long-term implant functioning is the modulation of immuno-inflammatory responses upon implantation. While titanium is considered ‘inert’ and does not result in an ‘adverse’ reaction, modulation of the host’s immune response (e.g., foreign body response) remains an emerging research avenue.^53^ Foreign body response is dictated via protein adsorption, acute/chronic inflammatory phases, foreign body giant cell formation and fibrous encapsulation upon implantation surgery at the implant’s surface.

In fact, the surgical placement of titanium implants at the bone site causes unavoidable local trauma and activates the innate immune system (that aims to counter injury and prevent infection). Despite sterile surgical procedures, the damaged tissue releases immuno-promotive cues (e.g., damage-associated molecular patterns) that can exacerbate inflammation.^54^ At the injury site, first, the neutrophils are recruited, followed by macrophages 1–3 days later (macrophage differentiates from the circulating monocytes).

The field of ‘osteoimmunology’ is emerging and has established the link between the immune and skeletal systems, and this knowledge derived from fracture healing investigations also extends to bone tissue trauma upon implantation surgery. The initial inflammatory phase is crucial for bone healing.^55^ During this phase, inflammatory cytokines and growth factors’ local production enable the bone formation and cell chemotaxis towards bone healing.^56^

Implant science and advances have been focussed on generating immunomodulatory surfaces so that a balance between inflammation and wound healing can be attained. For instance, use of specific nanotopographies that tune the macrophage polarisation from proinflammatory M1 to reparative/wound healing M2.^53^ Additionally, biomimetic approaches have also been employed to achieve favourable immunomodulation via micro- and nano-engineered implant surfaces.^57–59^ These biomimetic nanoscale implants enable regulating immune-inflammatory responses and achieving favourable immunotherapy and wound healing.^60^

It is well-established that an implant’s surface topography influences cellular responses.^61^ As a result, specific micro- and nanoscale topographical modifications have been performed on craniofacial metal-based implants to alter multiple cell functions.^62–64^ This knowledge has driven research into advancing implant surface modification techniques to enable superior bioactivity and bactericidal performances, especially in compromised conditions (poor bone quality/quantity).

## Nano-engineering of craniofacial titanium implants

Since the last decade, surface modification of implants to orchestrate osteogenesis has gained attention, encompassing various topographical, chemical or therapeutic modifications.^65,66^ Further, the extent of osseointegration depends on implant characteristics like roughness, wettability and chemistry that regulate the interaction between the implant surface and the bone. Various strategies modify the implants’ surface, augment the bone formation rates, and significantly reduce the time before implant loading.^67^ Evidenced by clinical success, surface engineering of implants via acid etching, silica/alumina particle blasting, CaP modification, and ion implantation have been showcased as ideal implants.^52^ More recently, nanostructuring of craniomaxillofacial and dental implants has emerged, as reviewed by Souza et al.^68^

Laser modification has been utilised to fabricate nanostructures on implants, with precise control over the geometry of nanotopographies.^69^ Laser treatments include laser-ablation and laser-induced surface melting that etches the implant surface to form nanoscale pits or grooves.^69–71^ Nd:YAG laser beam is the preferred laser source for modifying implants, attributed to their high energy density and the capacity for quick processing metals.^69–71^ Hallgren et al. fabricated hemispherical pits on implant surfaces with distinctive micro-nanoscale roughness. The modified implants showed improved osseointegration within rabbits’ tibiae at 12 weeks in vivo with increased bone-implant contact (BIC) area.^70^ Faeda et al. also reported similar results, that the Ti implants modified by Nd:YAG laser acquired increased BIC area within rabbit tibiae with significantly increased removal torque.^71^ Besides fabricating distinctive nanotopographies, the heat generated by laser treatment could thicken the TiO_2_ barrier layer on Ti implants, which improved their mechanical and chemical stability against corrosion.^72^ Although the laser modification could enable nanotopographies on the implant surface for improved osseointegration, the laser-modified nanostructures were restricted to the scale of hundreds of nanometres, which may be suboptimal for facilitating mechanotransduction towards further enhanced cell functions.

Alkali-heat (AH) could also facilitate a titanate layer with nanopillars on Ti implants that involves heating (400–600 °C) and immersion in an alkaline solution (mainly NaOH).^73,74^ Oh et al. reported that the titanate layer on AH-treated implants significantly enhanced the hydroxyapatite (HA) deposition within simulated body fluids, which improved osteocyte functions and promoted calcification and bone regeneration.^73^ Similarly, Krenek et al. reported significantly enhanced HA sedimentation on AH-treated Ti implants.^74^ Further, the distinctive nanopillars on the titanate layer also stimulated the human mesenchymal stem cells (MSCs) by significantly enlarging their spreading area and increasing their in vitro Alizarin expression at 5 days.^74^ Besides improving the osteoblasts’ functions, the fabricated nanostructures by AH-treatment could also inhibit the post-surgical inflammatory responses of craniofacial implants.^75^ As Liu et al. reported, on AH-treated Ti implants with nanoflakes and nanospikes, more filopodia and lamellipodia were stretched from RAW 264.7 macrophages, and their anti-inflammatory polarisation was promoted.^75^ However, the binding strength of AH-treated nanopillars with the underlying titanate layer might be suboptimal and easy to delaminate; hence the long-term mechanical stability of such nanostructures should be further investigated before their clinical translation.

An alternative strategy for implant nano-engineering includes depositing nanoparticles (NPs) that could mechanically stimulate osteoblasts and MSCs for improved osseointegration.^76,77^ As Areva et al. reported, a TiO_2_-SiO_2_ nanoparticular layer could be established on Ti implants by the sol-gel deposition technique.^76^ Briefly, the TiO_2_ and SiO_2_ sols were mixed with different ratios to combine into TiO_2_-SiO_2_ sols, followed by immersion of Ti implants and heat treatment to obtain a hybrid layer.^76^ The formation of TiO_2_-SiO_2_ NPs with varied TiO_2_ and SiO_2_ ratios showed different bioactivity enhancements. Further, the coating layer with more TiO_2_ NPs increased the early-stage proliferation of osteoblasts (7 days) and fibroblasts (3 days), contributing to enhanced wound healing.^76^ However, the coating with more SiO_2_ NPs augmented the alkaline phosphate (ALP) expression from osteoblasts (till 21 days), augmenting bone regeneration.^76^ Similar results were observed by Greer et al., that depositing TiO_2_ NPs on Ti implants via sol-gel technique yielded a particular nanoscale layer with distinctive surface roughness to stimulate the MSCs for enhanced osteocalcin (OCN) and osteopontin (OPN) expressions.^77^ This could be attributed to the topography of deposited NPs that mechanically stimulated the MSCs for enhanced osteogenicity, and was further supported by the in vivo results that improved OCN, OPN, ALP and collagen I were secreted from MSCs around modified Ti surfaces at 28 days.^77^ However, the adhesion of deposited NPs with the underlying implant surface could also be mechanically challenged to release the NPs into the surrounding tissues and raise toxicology concerns. Hence, investigations on evaluating and enhancing the mechanical stability of deposited NPs on modified implants are needed.

While various strategies, including mechanical, chemical, physical and biological techniques, have been utilised to impart nanoscale roughness/features to Ti implants, electrochemical anodisation (EA) is emerging to fabricate controlled titania (TiO_2_) based nanostructures on Ti implants.^78,79^ Briefly, EA involves an electrochemical balance between the formation and dissolution of TiO_2_ in an electrochemical cell (target Ti as anode, electrolyte containing water/F) with adequately supplied voltage/current.^80^ Under optimised conditions, the self-ordering of titania nanotubes (TNTs) or nanopores (TNPs) occurs on the implant’s surface. Additionally, by tailoring EA parameters, various nanostructures such as TNTs, TNPs and nanotemplates could be fabricated on the micro-rough implants to generate dual micro-nano topographies.^81,82^ Finally, the hollow nanostructures such as TNTs and TNPs could be utilised as reservoirs to load and deliver therapeutics into the surgical sites, promoting tissue regeneration, inhibiting bacterial invasion and achieving immuno-modulation around modified implants.^83^ Overall, anodised titanium-based implants demonstrate great potential towards fabricating bioactive and therapeutic craniofacial implants.

## Tailored therapy from anodised nano-engineered Ti implants

**Fig. 2:**
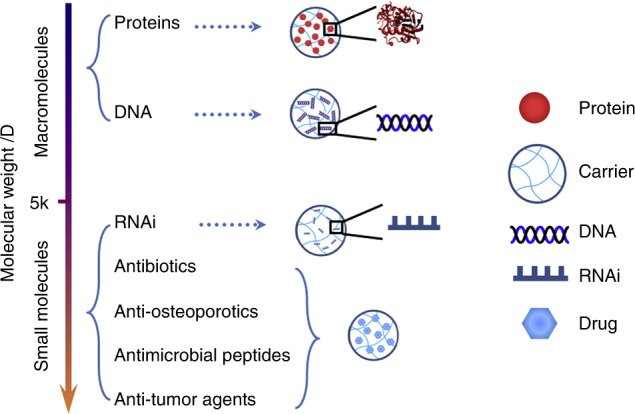
Classification of therapeutic molecules towards craniomaxillofacial bone regeneration. Adapted with permission from ref. ^84^

Local delivery of active biomolecules from synthetic biomaterials, scaffolds and implants for craniomaxillofacial bone regeneration holds great promise, as reviewed by Ji et al.^84^ As a result, local elution of such therapeutic agents directly at the defect site to induce bone regeneration may be an ideal approach, bypassing systemic administration and defect filling with grafts. Such biomolecules can be divided into large (cytokines, growth factors) or small (peptides, drugs, oligonucleotides) molecules based on their molecular weight (Fig. 2).^84^ Notably, the fragile nature of these molecules poses challenges to finding an appropriate delivery system that would allow for maximum therapeutic efficacy (controlled and sustained release) while maintaining the functionality of these molecules.^85^

### Bone therapy

#### Growth factor delivery towards osseointegration

Osteoblasts are primary cells involved in protein synthesis, growth factor regulation, mineralisation and recruitment/functioning of osteoclasts.^86^ Hence, the ability of an implant surface to modulate osteoblast activity (attachment, proliferation and differentiation) towards augmenting bone-implant integration is crucial to reduce clinical healing. Compromised conditions, including osteoporosis and diabetes mellitus, can present significant challenges to wound healing at the implant site and the extent of osseointegration.^87,88^

Growth factors (GFs) are biologically active molecules (mainly proteins and peptides) capable of stimulating tissue regeneration via stimulating growth, differentiation and activity.^89^ Administering via the systemic route to achieve osseointegration at the implant site has several shortcomings, including denaturation of sensitive proteins, inadequate local concentration, exposing the entire body to potent therapeutics and repeated hospital visits (poor patient compliance). To achieve repair and wound healing post-implantation and orchestrate osteogenesis, local elution of GFs from the implant surface allows for GFs retention and cellular uptake.^90,91^ For craniofacial regeneration (both hard and soft-tissue), achieving a local sustained release of potent growth factors via gene or cell therapy offers great potential, as reviewed elsewhere.^92^ For dental implants, appropriate osseointegration is crucial to early stability and long-term success, especially in compromised conditions with poor bone quality/quantity. As a result, to augment implant bioactivity, surface modification of Ti, Zr, TiZr and Ti-6Al-4V-based dental implants using various mechanical, physical, electrochemical and biomolecule modifications have been performed.^65,93^ Ti implants modified with TNTs offer the exceptional capability to enhance osseointegration abilities, attributed to ease of loading/releasing orthobiologics and established in vivo investigations.^94^ It is noteworthy that TNTs (as compared to micro-rough clinically used Ti) is osteogenic due to nanoscale roughness, mechanical stimulation of osteoblasts and incorporation of fluoride ions (during anodisation).

Various studies have utilised GFs coating on implants for enhanced bone regeneration, including bone morphogenetic protein (BMP), platelet-derived growth factor BB (PDGF-BB), vascular endothelial growth factor (VEGF), etc.^90,91^ BMPs are multi-functional growth factors that modulate bone regeneration and repair; numerous BMPs, including BMP-2, BMP-6 and BMP-4, have been immobilised on Ti-based implants to enhance bone regeneration and integration.^90,91^ To effectively improve the binding of BMPs on implants, Puleo et al. utilised the polymerisation method to deposit amino groups as a linker to connect BMP-4 with the underlying Ti implants.^95^ The plasma polymerised implants could effectively bind with the BMP-4 molecules, and the loaded surface significantly improved the ALP activity of the pluripotent stromal cells.^95^ Additionally, 3,4-dihydroxy-l-phenylalanine (DOPA) and dopamine were used to pre-treat Ti implants, increasing their affinity for immobilising BMP-2.^96^ Kang et al. reported that dopamine pre-treatment obtained a high number of amino groups on Ti implants than DOPA-treated counterparts and enabled higher BMPs adhesion via covalent bonding.^96^ The DOPA-BMPs coated surface significantly increased the ALP activity of C2C12 osteoblasts until 10 days, indicating the sustainable release of BMPs that augmented the osteogenic potential of the modified surface.^96^ An alternative solution is fabricating a porous surface by pulsed laser deposition for effectively loading BMPs. Briefly, a porous HA layer was coated on Ti implants (Ti-HA) via pulsed laser deposition, which could effectively load BMP-2, BMP-6 and BMP-7 by immersing into the respective solutions.^91^ Compared with the non-loaded Ti-HA counterparts, the Ti-HA-BMPs surfaces significantly promoted the osteogenic efficacy from C2C12 murine osteoprogenitor cells.^91^ Moreover, BMP-2 could significantly improve the early-stage osteogenic gene expressions towards improved bone healing, while BMP-6 inhibited the paracrine effects from C2C12 cells, which enabled a favourable osteogenic response.^91^

**Fig. 3:**
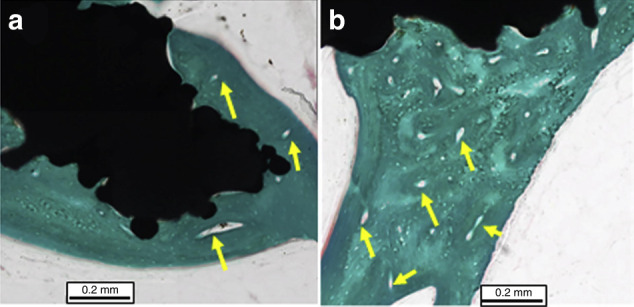
Growth factor releasing implants towards osseointegration. The histological sections of peri-implant bone around HA and VEGF-HA modified Ti implants. Optical micrographs of histological sections showed significantly thicker trabecular around VEGF-HA loaded Ti implants (**b**) than HA-coated counterparts (**a**) at 12 weeks within sheep tibiae. The yellow arrows indicate the newly formed blood vessels abundant around VEGF-HA loaded Ti implants. Reproduced with permission from ref. ^99^

VEGF has also been incorporated into Ti implants to enhance osteoblasts’ activity and improve the angiogenesis of surrounded endothelial cells.^97^ Guang et al. reported that VEGF-loaded Ti implants significantly promoted osteoblasts proliferation and augmented the expression of VEGF, ALP and Runx2.^97^ Further, in vivo results showed that the VEGF-loaded Ti implants adhered more OCN- and CD31-positive cells, indicating their enhanced angiogenesis potential.^97^ Similarly, Hu et al. immobilised VEGF on Ti implants via the cross-linking of carboxymethyl chitosan (CMCS) and hyaluronic acid-catechol (HAC).^98^ Compared with Ti-CMCS and Ti-HAC without VEGF loading, the VEGF-loaded counterparts were more hydrophilic and significantly promoted the proliferation of MC3T3-E1 cells on 4–7 days.^98^ Moreover, the ALP activity and calcium deposition from osteoblasts were also higher on VEGF-loaded implants, indicating their favourable osteogenic capabilities.^98^ The enhanced bone regeneration and angiogenesis on VEGF-loaded implants were also supported by the in vivo results, which enhanced new bone formation around VEGF-silicon-hydroxyapatite-loaded Ti implants (Ti@SiHA-VEGF) within sheep tibiae for 6 months.^99^ Further, the histological sections showed significantly thicker trabeculae with more blood vessels infiltration within the bone around Ti@SiHA-VEGF implants at 12 weeks, indicating the enhanced angiogenesis of VEGF-loaded Ti implants for accelerating bone regeneration and healing (Fig. 3).^99^

PDGF-BB improves osteoblast proliferation and osteogenesis. Keceli et al. reported the dissolution of PDGF-BB into silk fibroin solutions, followed by immobilisation on nanotubular Ti implants via electrical spinning of PDGF-containing silk fibroin.^100^ The PDGF-SF-coated Ti implants released PDGF within 7-14 days and significantly enhanced the expression of ALP and RUNX2 genes from MC3T3-E1 osteoprogenitor cells.^100^ Further, the mineralisation from MC3T3-E1 cells was improved considerably on PDGF-SF coated Ti implants between 14-28 days, indicating their influence on enhancing early-stage bone regeneration.^100^ Similarly, Hezaime et al. reported that PDGF-BB-coated Ti implants enhanced osseointegration, as confirmed by significantly increased BIC area, from the in vivo results within dogs’ mandibles at 3 and 6 weeks after implant placement.^101^

Platelets containing GFs are emerging in tissue regeneration applications attributed to their role in cellular migration, differentiation and angiogenesis.^102,103^ Venous blood is centrifuged at various speeds (with/without thrombin or anticoagulant) to yield platelet concentrates or autologous platelet concentrates (APCs). APCs contain GFs such as VEGF, insulin-like growth factor-1 (IGF-1), fibroblast growth factor (bFGF), PDGF-BB and transforming growth factor β-1 (TGF-β1). APCs, including platelet-rich plasma (PRP), platelet-rich fibrin (PRF) and concentrated growth factor (CGF), contain high levels of GFs and hence have been used in wound healing and tissue regeneration.^103,104^ PRP is produced via double centrifugation, PRF is platelet aggregation fibrin-rich gel obtained via single centrifugation of venous blood, and CGF contains a higher amount of GFs (better regeneration potential) than PRF and is obtained via varied centrifugation speeds.^104^

Besides coating GFs on implants, local application of GFs could also be obtained by grafting CGF at the implant site.^101,105,106^ CGFs are highly flexible to be placed in different wounded sites, enabling augmentation of bone healing at the implant sites and guided bone regeneration.^101^ Leucocytes were also detected from the CGFs, which could alleviate the post-surgical inflammatory responses when inserted in implantation and sites of guided bone regeneration.^105^ Based on these advantages, CGFs are promising therapeutic agents to improve treatment outcomes associated with implantation and bone augmentation surgeries.

#### Bone regeneration inhibition (craniosynostosis)

Craniosynostosis affects one in 2 500 children and involves premature fusion of one or more cranial sutures, resulting in extreme complications.^107^ Cranial vault reconstruction surgery is performed to release synostosed suture and correct craniofacial deformities; however, rapid bone growth at the surgical site represents a clinical challenge. The repeated interventions to cater to growing brains and potential morbidity and complications have driven the research for alternative molecular therapies.

Recent advances have highlighted the pathophysiology of craniosynostosis and the identification of biochemical pathways that influence normal/pathological morphogenesis.^108,109^ While surgery remains the primary treatment option for craniosynostosis, an improved understanding of the biomolecular mechanisms involved in suture fusion can yield novel strategies to achieve effective therapy. It is known that TGF-β (transforming growth factor) family members such as BMP-2 (bone morphogenetic protein) are primary bone inducers, and any mutation in the BMP signalling can be linked to craniosynostosis.^110^ Hence, manipulation of BMP pathways can be used to correct craniofacial anomalies. Controlling the bone growth rates via the downregulation of BMP osteogenic activity, bone-antagonising proteins like glypicans (GPCs) and noggins hold significant therapeutic potential.^109,111^ Investigations have supported that local delivery of GPCs can improve treatment outcomes via regulating skull growth.^111^ Towards maximum therapeutic efficacy, GPC local therapy’s challenge is achieving long-term sustained and controlled local elution at the designated site.^112^

Local elution of specific biomolecules combined with the influence of altered topography and chemistry of nano-engineered implants holds excellent promise to bypass the need for multiple surgeries in craniosynostosis by reducing bone re-ossification. Bariana et al. have explored the influence of the release of biomolecules from TNTs-modified implants towards enhanced craniosynostosis therapy.^113–115^ In 2017, the authors utilised large-diameter TNT implants loaded with/without biopolymer modification (Chitosan and Pluronic-F127) and evaluated the response of human suture mesenchymal cells (extracted from patients undergoing craniofacial reconstruction surgery).^113^ The TNT implant morphology reduced suture mesenchymal cell adhesion and proliferation, and both polymer modifications decreased cell morphology and function, indicating the suitability of the implant system to reduce sutural bone growth in advanced craniosynostosis therapy.

**Fig. 4:**
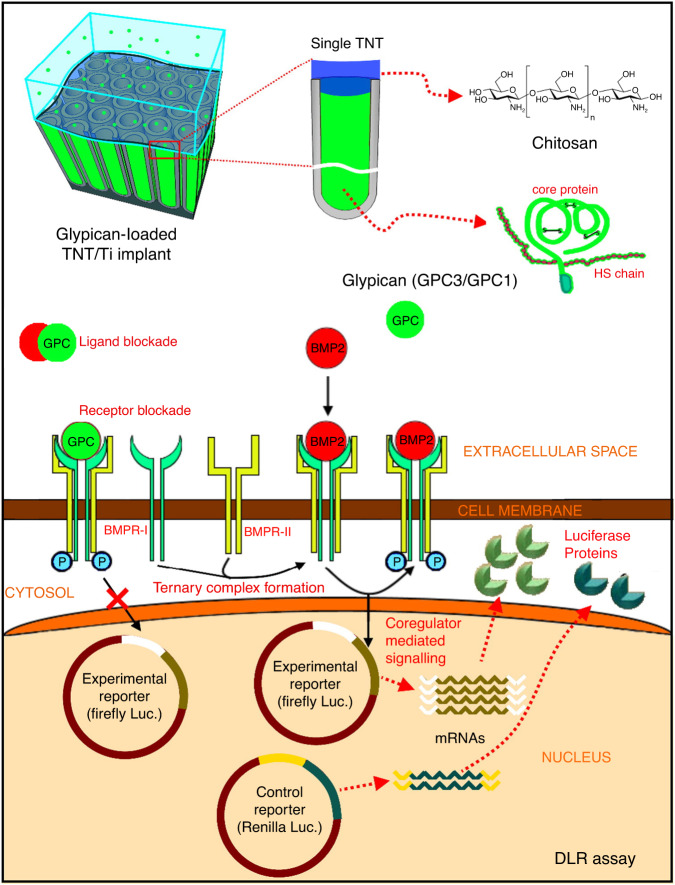
Nanotubular implants towards craniosynostosis therapy. Schematic representation of titania nanotubes (TNTs) modified Ti implants towards craniosynostosis therapy. Local controlled release of glypicans (GPC) and its influence on bone morphogenetic protein 2 (BMP2) signalling pathway via dual luciferase reporter (DLR) assay. Adapted with permission from ref. ^114^

In 2018, Bariana et al. used TNTs-based implants to load glypicans 1 and 3 (GPC1 and GPC3, which reduces BMP2 activity in prematurely fusing sutures) for its local elution (Fig. 4).^114^ Briefly, bare TNTs were loaded with therapeutics followed by a coating of chitosan to delay initial burst release (IBR) and have a sustained, controlled release. The released glypicans were tested for BMP-2 activity regulation in C2C12 murine myoblast cells in vitro. Chitosan coating on TNTs reduced IBR from ~63 to 36%, while 100% release was delayed from 4–5 days to 14–16 days. Assays confirmed that the released GPC1 and GPC3 maintained their functionality (during loading and release from TNTs) and maintained a reduction in BMP2 induction in transfected cells during their release.

In 2019, the same group tested the proof-of-concept glypican-releasing TNTs-based implants in vivo in murine models of Crouzon syndrome.^115^ In Crouzon murine model, surgical controls (without TNTs or protein) and TNTs loaded with GPC3 (with and without chitosan coating, as the experimental group) were implanted for 90 days. After retrieval, histological analyses confirmed that the eluted GPC3 from TNTs implants successfully inhibited bone regeneration in a craniosynostosis model. This implant model was proposed to improve treatment outcomes in affected children by reducing challenges associated with repeated cranial vault reconstruction.

### Soft-tissue integration (dental implants)

Implant therapy has been a routine treatment for edentulous and partially edentulous patients with a high predictability of long-term outcomes.^116^ Based on Branemark’s concept of osseointegration, various micro- and nano-engineering techniques have been employed to modify the implant surface, improving wound healing and the establishment of osseointegration.^117^ Along with the efforts made above, the implant treatment philosophy has also shifted from restorative-driven to biological-driven implant therapy.^118^ Studies even proposed the method which combines ‘gold standard’ micro-roughness with nano-engineering to fabricate dual micro-nano implants to augment osseo- and soft-tissue integration (STI) and reduce the risk of peri-implant infections.^119,120^

Orchestration of osseointegration from implant surface engineering has been well-researched and clinically established. However, the formation and maintenance of the soft tissue seal around implants is also a critical determinant of implant success but remains poorly researched.^93^ Adequate soft-tissue integration (STI) between the implant neck and gingiva is an essential prerequisite for the long-term stability of the gingival margin and underlying crestal bone, operating as a protective barrier to prevent bacterial ingress.^118^ Peri-implant soft tissue seal comprises ~2.5 mm epithelia apparatus (sulcular epithelium and junctional epithelium) and 1–1.5 mm supracrestal connective tissue.^121,122^ Adherence of apical third junctional epithelium onto implant neck or abutment by hemidesmosomes acts as the first barrier of antimicrobial defence.^123^ Meanwhile, the supracrestal connective tissue can attach directly to the implant and abutment surface to protect the underlying bone.^123^ As surrounding soft tissue adapts to the peri-implant micro-environment, an appropriate adherence of the epithelial and connective tissue is essential for soft-tissue maintenance around implants that shield from the ever-present pathogenic bacteria.^124^

**Fig. 5:**
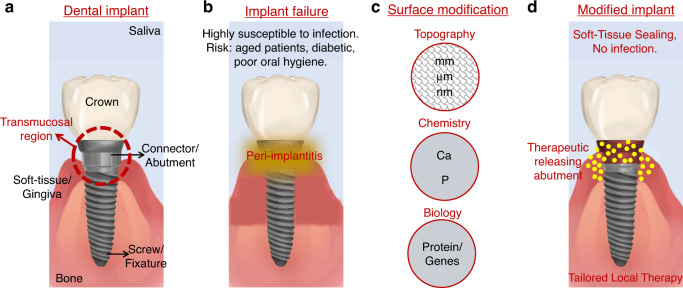
Drug eluting implants address dental implants’ soft-tissue integration (STI) challenges. Scheme showing **a** dental implant and transmucosal region; **b** peri-implantitis; **c** surface modification to achieve STI; and **d** next generation of modified therapeutic implants releasing active drugs/proteins directly inside the transmucosal region. Adapted with permission from ref. ^93^

Various investigations have established that varied roughness is required for the different parts of the implant to take advantage of the specific interaction features with the surrounding tissue. For instance, a smooth implant neck is proposed to prevent plaque accumulation and contamination.^125,126^ However, as mentioned above, to form a peri-implant soft tissue seal, there are also many aspects related to cell adhesion onto the implant neck, such as emergence profile design, micro- and nanoscale structure, surface chemistry and wettability.^118,127^ Among these, the application of nanoscale modification appears to be an effective method to enhance the epithelial and connective tissue responses, as reviewed recently.^93^ Various implant treatment strategies have been employed to fabricate nanostructures on the implant surface, including physical deposition, chemical etching, plasma treatment and anodisation, to augment STI.^65^ Figure 5 details the STI challenge in a dental implant setting, surface topography, chemistry and bioactivity modification and the emerging drug-eluting implants.^93^ Among the treatments above, anodised Ti implants with TiO_2_ nanostructures (like nanotubes or nanopores) have gained significant attention to orchestrate STI.^26^ The following sections detail influence of implant nanotopography and local drug release from anodised Ti implants towards specific cell modulation to achieve the timely establishment of STI.

#### Epithelial cells

Epithelial cell functions have been reported to be enhanced on nanostructured Ti surfaces, attributed to enhanced surface roughness/area and wettability.^128^ On nano-engineered Ti substrates, keratinocyte filopodia were guided by surface topographical features, representing a mechanotransduction mechanism of enhancing epithelial cell activities.^129^ Zhou et al. have reported that anodised Ti implants with 85 nm-diameter nanotubes could significantly promote epithelial cell adhesion and proliferation, but an adverse effect was detected when the diameter was reduced to 55 nm.^120^ In another study by Takebe et al., an anodised and thermally-treated Ti surface enhanced the proliferation and adhesion of gingival epithelia cells.^130^ Upregulation of integrin-α6β4 and laminin-5 (α3, β3, 2) mRNA expressions which are tightly associated with adhesion and proliferation of gingival epithelial cells has also been observed on hydrothermally-treated nanopores.^128^ Several surface coatings or therapeutic modifications have been superimposed on anodised nano-engineered Ti substrates to enhance the proliferation of human gingival epithelia cells.^93^ Xu et al. incorporated CaP NPs onto anodised micro-rough selective laser melted (SLM) Ti to augment the activity of human gingival epithelia cells.^131^ The results revealed that dual micro-nano CaP-anodised Ti significantly enhanced the adhesion, proliferation and expression of adhesion-related genes.

#### Fibroblasts

A significant amount of research has been performed to apply nano-scale modification to promote fibroblast activities. Enhanced proliferation and adhesion of human gingival fibroblasts were observed on titania nanostructures, including TNTs and TNPs.^125,132^ TNPs have been considered to have superior mechanical stability and may be an appropriate candidate for implant modification.^133^ Studies relating to the nanopores demonstrated the effect of fabricated nanopores on the upregulation of fibroblast activities and the promotion of cell-to-environment interactions.^134^ Gulati et al. fabricated unique anisotropic TNPs on micro-rough Ti surfaces via EA to preserve the underlying micro topography and generate dual micro-nano structures.^135^ The aligned dual micro-nano TNPs mechanically stimulated gingival fibroblasts and enhanced soft-tissue integration abilities.^135^

The study by Zhou et al. showed that Ti surfaces with 55 nm diameter nanotubes presented higher cell viability for initial fibroblast adhesion; however, no significant difference was found after cultivation for 3 days.^120^ Based on different cell behaviours, authors believed nanotube arrays could dramatically promote fibroblast adhesion; however, they inhibit its proliferation.^120^ Xu et al. also tested the performance of dual micro-nano CaP NPs loaded TNTs on HGFs and reported that the modification (anodisation and CaP NPs) exhibited the highest adhesion, proliferation and expression of adhesion-related genes.^131^ Next, Liu et al. incorporated bovine serum albumin (BSA) inside TNTs to enhance human gingival fibroblast functions.^136^ The results revealed that BSA-TNTs augmented human gingival fibroblast adhesion while suppressing late proliferation. To achieve rapid and firm soft-tissue sealing that protects the underlying implant structures against microbial ingress, Ma et al. showed the dual functionality of Ag NPs, and FGF-2-incorporated TNTs/Ti implants.^137^ Post anodisation, TNTs on Ti implants were electrodeposited with Ag NPs, followed by FGF-2 immobilisation via repeated lyophilization. Ag/FGF-2-TNTs showed favourable cytocompatibility with negligible cytotoxicity and increased attachment, proliferation and extracellular matrix-related gene expression of human gingival fibroblasts. Further, Wei et al. loaded TNTs with C-terminal CCN2 (connective tissue growth factor) fragments to achieve augmented STI ability from fibroblasts.^138^ Briefly, 80% loading efficiency was achieved for CCN2 loading into TNTs via the lyophilization method. Compared with Ti and bare TNTs, CCN2-releasing TNTs augmented the activity of human skin fibroblasts and exhibited enhanced actin cytoskeleton organisation.

### Antibacterial therapy

**Fig. 6:**
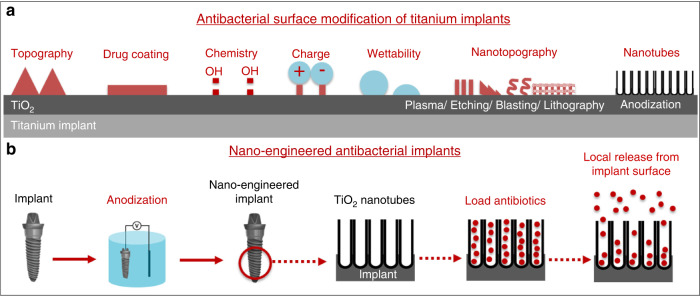
Antibacterial surface modification of Ti implants. Schematic representation: **a** various physical/chemical surface modifications; and **b** electrochemically anodised Ti implants with TiO_2_ nanotubes towards local elution of antibiotics. Adapted with permission from ref. ^140^

While nanoscale implant surfaces augment the activity of osteoblasts and other implant-relevant cells such as fibroblasts and epithelial cells (towards soft-tissue integration in dental implants), owing to the abundant area and nanoscale roughness, bacterial adhesion and biofilm formation may also be enhanced.^139^ Despite strict aseptic surgical insertion, bacterial infection is one of the major causes of implant failure, often resulting in the need for revision surgery and re-implantation. We have recently reviewed the influence of implant surface characteristics on bacterial adhesion and implant infection.^140^ Briefly, the implant infection cascade follows: (a) instant and reversible physio-chemical interaction; (b) irreversible molecular interaction; (c) formation/maturation of biofilm; and (d) shielded by biofilm, continued bacterial proliferation.^141^ In a recent review, Zhang et al. categorised various bactericidal strategies into tackling adhesion, colonisation, biofilm and proliferation, relating to the stage of infection.^139^ As a result of increased implant placement and risk of infection (especially in compromised patient conditions), implant research has been focused on fabricating the passive (physical/chemical modification) or active (ability to release therapeutics) implant surfaces.^66^ Figure 6 shows surface modification strategies employed to achieve antibacterial functions from nano-engineered Ti implants.^140^

#### Bioinspired implant nanotexturing

Mimicking naturally existing nanotopographies in wings of cicadas, moths and dragonflies and shark skin and gecko skin, various nanostructures (pillars, needles and spikes) have been fabricated to achieve high bactericidal action via the ‘bed of nails’ mechanism.^66^ To attain such nanotopographies, strategies like chemical etching, inductively coupled plasma reactive ion etching, hydrothermal, lithography and anodisation have been employed to modify Si, SiO_2_, polymers and metals (including stainless steel, Zn, Ti and its alloys^142^). The antibacterial efficacy of such nanotextures is dictated by their organisation (spacing, dimensions), hydrophilicity and the type of bacteria (Gram-positive or negative).^143^

Zhang et al. fabricated Sr-Ag co-substituted fluorohydroxyapatite (SrAgFHA) nanopillars/rods (100–150 nm) on Ti via electrodeposition that demonstrated improved corrosion resistance than FHA and bare Ti.^144^ Further, the study revealed that SrAgFHA showed the best osteogenic (MC3T3-E1 cells) and bactericidal (*S. aureus* and *E.coli*) effects in vitro and new bone formation in rabbit defect model in vivo. These performances suiting clinical implant applications were attributed to sustained ion release, nanoscale roughness and superhydrophobicity of the SrAgFHA coatings. Interestingly, F doping restricted the release of Ag+, thereby reducing any potential cytotoxicity.

**Fig. 7:**
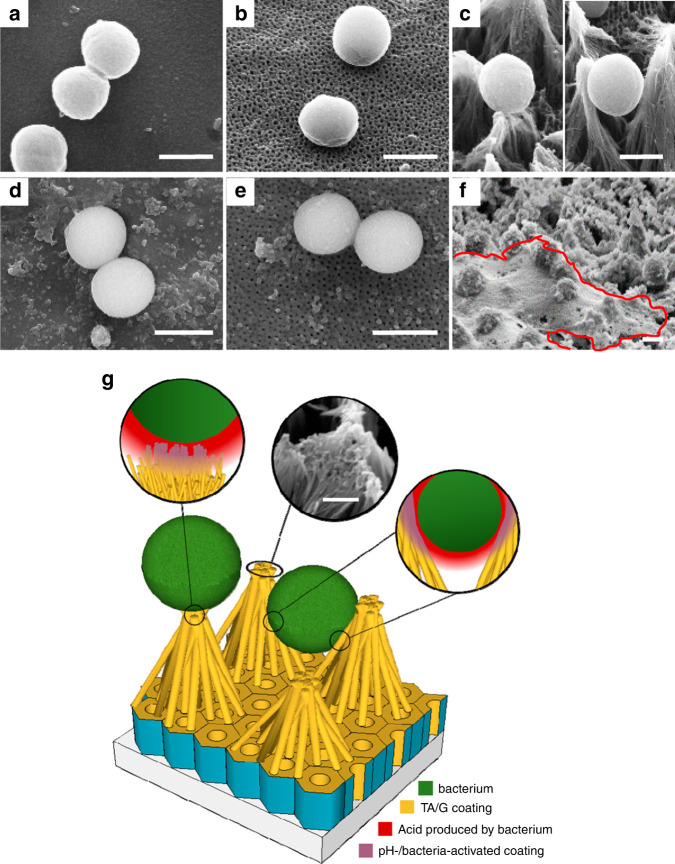
Bacterial adhesion on antibiotic-releasing nanopillar surface. SEM images showing adhesion of *S. aureus* on **a**, **d** smooth electropolished Ti; **b**, **e** 2D TiO_2_ nanopores; and **c**, **f** 3D TiO_2_ nanopillars surfaces, without (top-row) and with (bottom-row) gentamicin-tannic acid coating. **b** Shows bacterial attachment on 3D nanopillar top and gaps between pillar bundles. **f** The red marking indicates an extracellular polymeric substance excreted by bacteria. **g** Schematic representation of bacterial adhesion on tannic acid/gentamicin coated 3D nanopillars. Scale bar **a**–**f** represents 1 µm. Adapted with permission from ref. ^145^

Hizal et al. utilised electrochemical anodisation to fabricate 2D TiO_2_ nanopores and 3D nanopillars on Ti, followed by layer-by-layer self-assembly of tannic acid and gentamicin.^145^ The investigation of adhesion and growth of *Staphylococcus aureus* revealed that the coating of 3D nanopillars with tannic acid/gentamicin enabled a 10-fold reduction in the number of attached bacteria. This result was attributed to a combined effect of a large surface area for the local release of antibiotics and reduced antibiotic defence from bacteria due to reduced adhesion to the surface (Fig. 7). Further, analysis of adhesion forces between a single bacterium and modified surfaces via atomic force microscopy– single-cell force spectroscopy revealed that forces followed the trend: nanopillars < nanopores < polished.

#### Anodised therapeutic implants

Electrochemically anodised biomedical implants, including Ti and its alloys with controlled titania nanostructures like nanotubes, can be utilised to load and locally elute potent antibiotic agents (Fig. 6b). Bacterial adhesion and biofilm formation on TNTs/Ti implants is influenced by their diameter, wettability, and crystallinity, as reviewed elsewhere.^140^ The utilisation of the test-tube-like morphology of TNTs to tailor local therapeutic elution has been widely researched. It is worth noting that the open pores of the tubes can cause very high release rates as the implant is placed (attributed to diffusion gradient) that can cause local toxicity, referred to as IBR.^146^ Hence, the release must be tailored to reduce IBR and achieve a long-term sustained release pattern for optimum therapeutic performance. Potent antibacterial agents such as antibiotic drugs (gentamicin, vancomycin),^147^ antimicrobial peptides,^148^ biopolymer-assisted controlled release (using chitosan, silk, polydopamine, etc.),^149^ and doping with metallic NPs/ions (Ag, Au, Cu, Ga, Zn, etc.),^150^ have been incorporated inside/on TNTs-modified Ti implants to achieve effective bactericidal action, with favourable bioactivity.

In their pioneering attempt, Popat et al. in 2007 utilised gentamicin-loaded TNTs to investigate the influence of its local elution on osteoblast activity and antibacterial effectiveness.^147^ The study revealed that the favourable bioactivity of osteoblasts was maintained while a significant reduction in the adhesion of *S. epidermidis* was confirmed. In a dental implant-relevant study, tetracycline-loaded TNTs showed significant inhibition of early colonisation (<3 h) of *P. gingivalis*.^151^ Further, the release of tetracycline did not cause any toxicity to bone marrow stem cells.

It is noteworthy that with the emergence of antibiotic-resistant bacteria like methicillin-resistant *Staphylococcus aureus*, alternate therapeutic strategies are needed to combat implant-related infection. Ma et al. used antimicrobial peptides (AMPs) to achieve their local release from TNTs and observed ~99.9% bactericidal action against *Staphylococcus aureus*.^152^ Li et al. loaded GL13K (a protein in the AMPs family) on TNTs and observed significant inhibition of *F. nucleatum* and *P. gingivalis* without any toxicity to adjacent osteoblasts.^153^ Further, Zhang et al. fabricated dual-diameter TNTs with upper 35/75 nm diameters as nanocaps and underlying 140 nm diameter tubes as nanoreservoirs to accommodate AMP ponericin G1.^154^ Dual-diameter TNTs showed sustained release of AMP for up to 60 days (conventional one-diameter TNTs only up to 42 days) and showed both short- and long-term (up to 49 days) antibacterial efficacy against both planktonic bacteria (release-killing from AMPs) and adhered bacteria (AMP-derived killing and nanocap driven adhesion resistance).

Biopolymers like chitosan, polydopamine and PLGA [poly(lactic-co-glycolic acid)] have also been utilised to control the release of antibiotics and impart enhanced bioactivity.^149,155,156^ To achieve optimum bactericidal efficacy, in separate experiments, potent antibiotic agents, including Ag NPs^157^ and Zn-Ag NPs,^155^ were loaded inside TNTs using polydopamine. The results revealed trap-killing of bacteria and dual antibacterial/cytocompatibility functions, respectively. Next, gentamicin-releasing TNTs showed superior antibacterial/antibiofilm abilities while enhancing osteoblast adhesion in vitro.^158^ Further, Fathi et al. used electrospun silk fibroin nanofibers on TNTs preloaded with antibiotic vancomycin and reported high bactericidal efficacy against *S. aureus*.^159^

Antibacterial metal-based ions and NPs, including Ag, Cu, Zn, Ga, Au, etc., have also been utilised to achieve antibacterial functions from TNTs-modified Ti implants. Ag ions or NPs have been most widely researched to modify TNTs and aim to strike a balance between antibacterial, bioactivity and cytotoxicity functions.^150,160,161^ Various methods have been utilised to incorporate Ag ions or NPs inside TNTs for their local elution, including spin-coating,^160^ chemical reduction,^150^ photo-reduction,^162^ micro-arc oxidation,^157^ magnetron sputtering,^163^ electrophoresis,^164^ and immersion/anodisation in AgNO_3_ solution.^161^ These investigations reveal that the local release of Ag ions/NPs from TNTs can enable high antibacterial efficacy while maintaining biocompatibility.

Xu et al. incorporated Au NPs inside TNTs and reported antibacterial efficacy against *P. gingivalis* and *F. nucleatum* upon ultraviolet irradiation and favourable immunomodulatory functions.^165^ Wang et al. used magnetic sputtering to dope TNTs with Au NPs, showing high antibacterial efficacy against *S. aureus* (with no reactive oxygen species/reactive oxygen species produced from NPs in the dark).^166^ Multiple NPs have also been incorporated inside TNTs/Ti implants to achieve combination therapies. For instance, Roguska et al. modified TNTs with Ag and Zn NPs via direct current magnetron sputtering to perform dual antibacterial and antifungal actions against *Candida albicans, Candida parapsilosis*, and *Streptococcus mutans*.^167^ The results revealed that dual Ag-Zn release eliminated bacteria within 3 h and fungus within 24 h contact.

Additionally, Xiang et al. used folic-acid conjugated ZnO quantum dots with vancomycin^168^; and Dong et al. used Ga-doping with (poly-DL-lactic acid)^169^ to achieve a significant reduction in bacterial adhesion with favourable biocompatibility. Very recently, Jayasree et al. showed superior bactericidal functions of Ga-doped dual micro-nanoporous Ti implants.^170^ Briefly, Ga-doping formed nanoscale particles firmly attached to the nanopores that exhibited significant antibacterial efficacy in the human oral salivary biofilm model. At the same time, it maintains favourable bioactivity for gingival fibroblasts. While the use of metal ions and NPs show significant antibacterial functions, it is noteworthy that the dose-dependent antibacterial behaviour can also cause local cytotoxicity; hence, it must be controlled.^171^

### Cancers and tumours

Head and neck cancer, including a group of malignancies occurring in the lip, oral cavity, pharynx and larynx, accounts for 2.3–6% of all malignancies.^172^ Further, nearly 90% of all head and neck cancers are squamous cell carcinoma.^173^ In most of these cancers, the oncological treatment involves the oral cavity, including surgery, chemotherapy, radiotherapy, or a combination of therapies.^174^ Along with the advance of nanobiotechnology in cancer, nano-oncology is generating increasing interest attributed to its multifunctional capacity in diagnosis and treatments. The advent of nanotechnology has enabled the identification of predictive molecular changes in cancer early stage, and delivering contrast agents to pinpoint cancer spread. NPs and nanomaterials have aided in effective local therapy via the delivery of drugs or genes, precise tumour removal, photodynamic/thermal treatment and enhancement of radiation. Compared to conventional chemo, radio and immuno-therapies, nanomaterials-based cancer therapy is customisable to match patients’ tolerance and therapeutic needs combined with improved bioavailability, precise monitoring and maximised therapeutic efficiency.

Early detection of tumours is often critical to increasing survival rates. The current primary diagnosis for head and neck cancer is via imaging, including CT, MRI, ultrasonography and PET. However, such imaging methods are insufficient to detect small lesion or early-stage cancers. Biopsy or needle aspiration can only be used after detecting cancers.^174^ Since the last decade, cancer diagnostic has been developed towards nano-scale detection that offers identification of associated biomarkers. Various nanostructures have been introduced with varying sizes, compositions (Au, Ca, iron oxide, liposomal) and shapes (NPs, nanotubes, nanocages, nanoshells, branched dendrimers, nanowire and polymeric).^175,176^

Head and neck cancer treatments include surgical resection, radiation therapy, and chemotherapy. When advanced tumours spread beyond the surgical margin, surgical resection will carry risks of involving adjacent critical anatomical structures. Ionising radiation is constrained by its toxicity. Challenges encountered by drug and chemotherapy include nonspecific distribution of anti-tumour agents and inadequate drug concentrations in the tumour. With the benefit of nanotechnology, selective delivery devices have been developed to load drugs or agents to tumour sites to increase delivery effectiveness and decrease toxicity. Also, current nanotechnology provides solutions to monitor posttreatment responses.

#### Therapy from nanoparticles (targeted and triggered)

Many kinds of NPs have been developed to deliver to the targeted cells easily and efficiently. Drug encapsulation can be achieved by integrating into the matrix or attaching to the surface of NPs, thus protecting the body against the toxicity of drugs. Angiogenesis is an essential factor for tumour growth and metastasis. Blood vessels recruited to supply tumour cancer with nutrients have 600–800 nm gaps between endothelial cells.^177^ Due to size and surface properties, NPs can extravasate through these gaps into vascular gaps and accumulate inside tumour sites. When coating with specific molecules binding to antigens or receptors on the tumour cell, drug distribution can be further enhanced in the cancer sites. After reaching the targeted region, drug release occurs for therapeutic purposes. Recent progress has also been made to conjugate monoclonal antibodies, plasma proteins and viral vectors for gene therapies.^178^ Different NPs have been investigated for delivery purposes, including liposomes, polymers, metal NPs, etc. Among them, Au NPs were the most compelling one as they exhibit the capability of selective delivery of large doses of toxins and enhanced properties of filtration and retention into tumours. Paciotti et al. demonstrated polyethylene glycol coated Au NPs attached with tumour necrosis factor rapidly and accumulated in MC-38 colon carcinoma tumour-bearing mice with little retention in other cells.^178^ Based on the composition and properties of NPs, many delivery devices have been developed to convey drugs, agents, genes and cells to tumour sites. Nano-fibre scaffolds were first investigated to deliver the drug to the target region.^179^

NPs can be used as imaging contrast agents to produce exceptional images of tumours because of their unique magnetic, electronic, photothermal or catalytic properties.^174^ Due to their small size (<100 nanometres) and high surface area to volume ratio, NPs can identify tumour cells via attachment of functional groups and selectively accumulate inside many tumour beds. The optical properties of noble metal NPs allow for enhanced spectral signals to be detected against the background of cells and extracellular tissue. Of currently available NPs, Au NPs are promising and widely used in detecting head and neck cancer because of their physicochemical and biological properties and relatively low toxicity. When light stimulates metal NPs, light scattering and light absorption occur. The attached antibody or substance on Au NPs will cause a slight red shift of the peak frequency of light. Then the measurement of light absorption reveals the targeted cells’ characteristics and provides optical signals for molecular information. Huang et al. reported a red shift in light frequency when Au NPs were coated with EGFR (epithelial growth factor receptor) antibody.^175^ The authors also demonstrated that the amount of NPs attached to the malignant cell was six times more than the control cells. The results were consistent with the overexpression of EGFR on the tumour cell.^175^

As fluorescent semiconductor nanocrystals, quantum dots are as attractive as optical imaging agents because of their unique optical electronic and photophysical properties and biostability. When coated with specific substances to enhance the attachment to cancer cells, quantum dots can improve imaging of tumour sites under the initiation of ultraviolet light.^180^ When stimulated by light, Au NPs can show other optical characteristics, such as fluorescence. Au NPs can quench the fluorescence of bounded molecules. The study by El-Sayed et al. demonstrated that Au NPs quench cellular autofluorescence by approximately 15% when incubated or immuno-conjugated to cells. This effect can also be used to detect malignant cells.^181^

#### Anticancer therapies from anodised Ti implants

##### Local anticancer therapy via drug release

Test-tube-like nanotubes fabricated on Ti-based implants are a favourable candidate for achieving local anticancer therapy and also provide mechanical support and enhance local tissue healing. As a result, TNTs have been utilised to accommodate potent anticancer therapeutics towards their local elution.^182^ Kaur et al. investigated localised cancer therapy using a pre-clinical cancer model to test the anti-tumour efficacy of TNF-related apoptosis-inducing ligand (TRAIL) releasing TNTs/Ti wire implants.^183^ TNTs (9 µm length and 50 nm diameter) were fabricated on Ti wire implants to achieve a TRAIL loading capacity of ~12.63 μg per implant. Compared to PBS-loaded TNTs, TRAIL-TNTs exhibited significant breast cancer cell death in 2D and 3D cell culture models in vitro. Next, the implants were placed in a subcutaneous tumour model in the back of nude mice in vivo, and TRAIL-TNTs showed significantly reduced tumour burden.

Khoee et al. reported incorporating 5-fluorouracil (5-FU) inside TNTs followed by covering with a liposomal cap (soy lecithin, cholesterol and polyethylene glycol) to achieve anticancer therapy.^184^ The number of liposomal coatings controlled the release of therapeutic. Next, the exposure of various concentrations of 5-FU loading (3, 30, 100, 200, 300, 1500 and 3000 g/mL) from TNTs was evaluated by culturing HeLa cell line (cervical cancer origin). TNTs enabled effective local therapy that enhanced drug uptake and reduced cell viability and colony formation in HeLa cells. In 2021, Gulla et al. reported using quercetin-conjugated TNTs (TNT-Qu) to induce apoptosis in B16F10 melanoma skin cancer cells.^185^ As compared to TNTs (14.4%) and quercetin-alone (44.86%) treatments, TNT-Qu inhibited migration and significantly reduced apoptosis (60.29%) in melanoma cells. Further, TNT-Qu reduced reactive oxygen species and superoxide levels. Additionally, anticancer functions of TNT-Qu were attributed to augmented cleaved caspase-3 levels. In 2022, Zandvakili reported the influence of TNTs deposited, doped and coated with Ag on the cytotoxicity of breast cancer cells.^186^ Ag-modified TNTs were annealed to crystallise into the anatase phase, followed by culturing MCF-7 cells (human breast carcinoma) in vitro. The results revealed that negatively charged TNTs repelled cancer cells and interfered with their growth and proliferation, followed by damage to the cell membrane and killing of the cancer cells.

##### Dual anticancer and antibacterial functions

Selenium (Se) possesses anticancer characteristics; however, its systemic administration can cause side effects and toxicity, and as a result, its local elution from implants holds significant potential.^187^ To achieve this, Chen et al. deposited Se inside TNTs via electrodeposition followed by chitosan coating to control Se release and investigated the functions of both healthy and cancerous osteoblasts in vitro.^188^ Chitosan-Si-TNTs exhibited sustained release of Se for 21 days. Further, Se release augmented healthy osteoblast proliferation while selectively inhibiting cancerous osteoblast growth. Next, the modified implants were tested with *E. coli*, and long-term bactericidal functions were exhibited. This study showcases the potential of tailorable dual anticancer and antibacterial therapy from Se-releasing TNTs coated with chitosan.

Bilek et al. used chemically synthesised Se NPs to decorate TNTs to evaluate the antibacterial and anticancer properties of the modified implants.^189^ Briefly, gram-negative bacteria *E. coli*, cancerous osteoblasts like MG-63 cells and non-cancerous fibroblast NIH/3T3 cells were cultured on the substrates in vitro. Proportional to the surface density of NPs, the Se NPs releasing TNTs exhibited antibacterial and anticancer activity. However, deterioration of adhesion and viability of non-cancerous cells was also reported. The authors reported reducing NP surface density to minimise local cytotoxicity to healthy cells.

##### Photocatalytic/photoinduced cancer killing

Photodynamic/thermal therapy is based on the principle that light-activated microscopic particles may heat adjacent cancer cells to cause their apoptosis.^190^ Many nanomaterials have been used, including iron-, gold-, magnetic NPs and carbon nanotubes. Such a technique is appealing because it is non-toxic and noninvasive. Composed of core and metal NPs coatings, nanoshells can be stimulated by infrared light and destroy targeted cancer cells via thermal destruction, with a low risk of involving surrounding tissues.^191^ The tissue penetration depth of conventional photosensitizers is limited. Nanoshell-structured NPs could convert the light with strong tissue penetration to ultraviolet-visible light to stimulate the photosensitizers. Nanoshells coated with gold have been proven to be an effective tool in vitro^191,192^ and in vivo studies.^193^ West et al. have reported the application of 120nm-diameter nanoshells conjugated with gold kill cancer cells via photodynamic therapy in mice.^194^ Such nanoshell-assisted photothermal therapy is also investigated in clinical practice to treat refractory head and neck cancers.

In a pioneering attempt, Kalbacova et al. in 2008 reported photocatalytic killing of cancer cells via TNTs.^195^ Briefly, the cultured HeLa G cells were killed upon ultraviolet irradiation as an excitation source for photocatalysis. Further, the TNTs dimensions influence cellular adhesion and spreading. Shrestha et al. modified TNTs by incorporating magnetic Fe_3_O_4_ NPs into the TNTs via a permanent magnet. This was followed by a demonstration of the photocatalytic activity (photo-induced oxidation of Acid Orange 7 dye) via the shining of ultraviolet light.^196^ Next, upon movement of a magnet under the petridish containing modified TNTs, the TNTs moved in the direction of the magnet, confirming the magnetic conversion of TNTs. Further, a fluorescent marker (model drug) was attached to TNTs via a silane coupling agent to showcase drug-releasing ability. Upon ultraviolet irradiation, the release of the marker could be observed. Finally, ultraviolet-induces cancer cell killing was observed with HeLa tumour cells.

##### Magnetically-targeted cancer therapy

Magnetic nanosensor technology has a potential application for detecting cancer biomarkers in the bloodstream or other bodily fluids. Magnetic NPs have been developed to be an efficient approach to liquid biopsy due to the high binding efficiency and stability in most aqueous solutions of magnetic NPs.^197^ The technique involves the integration of multiplex targeting, magnetic enrichment, signal amplification and recognition.^198^ Magnetic NPs modified with anti-epithelial cell adhesion molecules have been proven sensitive and accurate for diagnosing metastatic breast, colorectal or prostate cancer via detecting CTC.^199^ Kafshgari et al. generated magnetic TNTs by incorporating a ferrofluid containing iron oxide NPs (diameter ~10 nm) in planar weakly-connected TNTs sheets.^200^ Post-annealing, the TNTs were loaded with camptothecin and cultured with HeLa cells. Findings show that internalisation of magnetic TNTs-based drug carriers was enhanced into the HeLa cells upon application of a static gradient magnetic field. While this proof-of-concept study demonstrates the use of magnetically-targetted TNTs, it is noteworthy that the TNTs used are loose and do not adhere to a Ti implant surface. Alternately magnetic field-triggered local therapy has also been demonstrated for TNTs/Ti implants, as reviewed elsewhere.^49^

## Clinical translation challenges

**Fig. 8:**
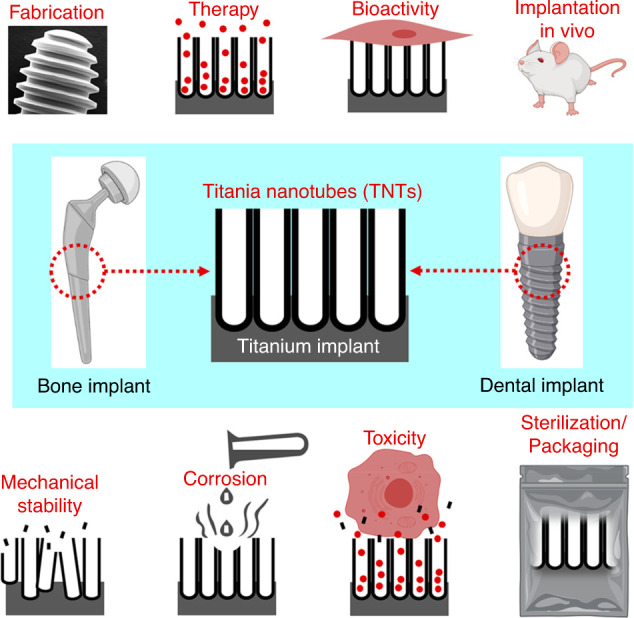
Clinical translation challenges associated with therapeutic anodised implants. Adapted with permission from ref. ^216^

The critical clinical translation challenges for drug-eluting nano-engineered craniofacial implants that need further investigation to ensure the transition from laboratory to benchside are summarised in Fig. 8. Nanotechnology offers a practical and broad approach to enhance tissue formation/integration, anti-bactericidal functionality and local therapy to treat tumours. Nevertheless, local cytotoxicity concerns remain unaddressed with respect to ions/ NPs accumulating within the local tissues. TiO_2_ NPs or loose TNTs could be released from anodised Ti implants due to electrochemical/chemical corrosion or mechanical damage caused by exposure to a specific microenvironment, which may induce toxicity, including oxidative stress, organ pathologies, respiratory distress, etc.^201^ Recent studies showed that TiO_2_ particles caused respiratory toxicity and epithelial inflammation of lung tissue in animal models.^202,203^ DNA damage and other adverse effects have also been reported due to potential NPs cytotoxicity. According to the study by Shukla et al., TiO_2_ NPs of 1 μg/mL could induce DNA damage and cause apoptosis in HepG2 cells.^204^ Hu et al. reported the most severe DNA damage observed at 10 μg/mL. Saquib et al. also found significant dsDNA damage when exposed to 20 μg/mL nano-TiO_2_ over 3 h.^205^

The toxicity of NPs depends on their crystalline characteristics, chemical composition, and surface charge. Hu et al. reported that 200 nm chitosan NPs could cause 100% mortality to the embryos and severe teratogenic deformities compared to 340 nm NPs.^206^ A study on the cytotoxicity of various crystalline structures revealed that anatase-TiO_2_ NPs exhibited spontaneous generation of reactive oxygen species from anatase form. According to the study by Valdiglesias et al., TiO_2_ NPs could induce cytotoxicity, genotoxicity, and oxidative damage in SHSY5Y cells.^207^ A mix of crystalline forms of nano-TiO_2_ (anatase and 80% anatase with 20% rutile) was comparable in inducing DNA strand damage.^207^ However, no toxic effect on the protozoan population of the nano-TiO_2_ was reported in another study.^208^ Oxidative stress has been considered a common mechanism for NP-induced cell damage.^209^ Antioxidant enzyme activity and total antioxidant capacity could decrease severe oxidative stress.^209^ While metal NPs (e.g. Ag, Zn, Cu, Zr and Si) offer local therapies, they may also cause dose-dependent cytotoxicity via the release of free ions.^171^ Hence, a thorough investigation of their bioactivity/therapy vs toxicity must be performed.

The craniofacial region, especially the oral cavity, is under constant masticatory loading, which can challenge the nanotubes’ survival. As a result, mechanical characteristics, including hardness, modulus and fracture strength of the implant and its modification, is essential. It is also noteworthy that an implant must survive mechanical forces experienced during handling, surgical placement and under-load application. Any mechanical mismatch with the surrounding tissue or onset of corrosion (due to a change in pH or infection) can cause cracking or delamination of the anodic film. Electrolyte aging (repeated use of the same electrolyte for anodisation before the target implant) has demonstrated favourable results in fabricating stable and well-adherent anodic film on implants.^210–212^ Further, single-step anodisation of micro-rough implants with aged electrolyte yields a dual micro-nanoporous surface with superior mechanical characteristics compared to conventional nanotubes.^133,213^ Besides, various chemical and physical enhancements have also been performed to augment the mechanical strength of nanotubes, as reviewed elsewhere.^133^ Unfortunately, there is a lack of investigation that tests the stability of anodised implants in long-term in vivo models and under mechanical (masticatory) loading. Further, corrosion and electrochemical instability pose challenges in the dental implant setting, so thorough examination of drug-eluting TNTs in corrosive environments is needed.

Soon after implantation of a drug-loaded implant, attributed to diffusion gradient, there is a sudden IBR that may account for a significant amount of the entire payload. While there are strategies to control the release kinetics (like capping or coating of drug-loaded nanotubes or drug encapsulation before loading^214^), IBR can cause local cytotoxicity and needs further examination. Various studies also showcase the potential for using metal-based ions or NPs or external triggers; however, their effectiveness in real compromised in vivo conditions has not been appropriately explored. Further, accidental triggers are at high risk for light/magnetic trigger-based release. Additionally, in vitro release does not directly translate into the compromised implant micro-environment in vivo, as often the local trauma and coagulation can impede the effective clearing of the drug, thereby significantly reducing the diffusion gradient.^215^

## Conclusions and perspectives

Bioactive and therapeutic electrochemically anodised titanium implants with nanotubes exhibit favourable characteristics that warrant their use to address craniofacial therapeutic challenges, including failing dental implants, suboptimal osseointegration in bone fixation, craniosynostosis and tumour treatment. Fabricated via a cost-effective and scalable anodisation technique, nanotubular titanium implants can accommodate the drug of choice and deliver it locally inside the craniofacial microenvironment in the desirable therapeutic window, enabling customisable local therapy. Various proof-of-concept investigations confirm the significant potential of local therapy from the implant surface to achieve timely therapeutic outcomes towards long-term implant success; however, clinical translation challenges remain unaddressed. Cytotoxicity, lack of long-term in vivo investigations, therapeutic response under mechanical loading in compromised tissue and long-term mechanical stability are the key hurdles to overcome. The future of craniofacial therapy includes advanced nano-engineered implants that provide mechanical support and orchestrate tissue integration while simultaneously enabling the delivery of potent therapeutics to achieve accelerated tissue regeneration/correction.
